# A Thiourea Derivative of 2-[(1*R*)-1-Aminoethyl]phenol as a Chiral Sensor for the Determination of the Absolute Configuration of *N*-3,5-Dinitrobenzoyl Derivatives of Amino Acids

**DOI:** 10.3390/molecules29061319

**Published:** 2024-03-15

**Authors:** Federica Aiello, Alessandra Recchimurzo, Federica Balzano, Gloria Uccello Barretta, Federica Cefalì

**Affiliations:** 1 Institute for Chemical and Physical Processes, National Research Council, Via G. Moruzzi 1, 56124 Pisa, Italy; federica.aiello@cnr.it; 2 Department of Chemistry and Industrial Chemistry, University of Pisa, Via G. Moruzzi 13, 56124 Pisa, Italy; alessandra.recchimurzo@phd.unipi.it (A.R.); f.cefali@studenti.unipi.it (F.C.)

**Keywords:** NMR, chiral solvating agents, sense of nonequivalence, enantiodiscrimination, chirality

## Abstract

In the exploration of chiral solvating agents (CSAs) for nuclear magnetic resonance (NMR) spectroscopy designed for the chiral analysis of amino acid derivatives, notable advancements have been made with thiourea–CSAs. **1-TU**, derived from 2-[(1*R*)-1-aminoethyl]phenol and benzoyl isothiocyanate, is effective in the enantiodifferentiation of *N*-3,5-dinitrobenzoyl (*N*-DNB) amino acids. In order to broaden the application of **1-TU** for configurational assignment, enantiomerically enriched *N*-DNB amino acids were analyzed via NMR. A robust correlation was established between the relative position of specific ^1^H and ^13^C NMR resonances of the enantiomers in the presence of **1-TU**. 1,4-Diazabicyclo[2.2.2]octane (DABCO) was selected for the complete solubilization of amino acid substrates. Notably, the *para* and *ortho* protons of the *N*-DNB moiety displayed higher frequency shifts for the (*R*)-enantiomers as opposed to the (*S*)-enantiomers. This trend was consistently observed in the ^13^C NMR spectra for quaternary carbons bonded to NO_2_ groups. Conversely, an inverse correlation was noted for quaternary carbon resonances of the carboxyl moiety, amide carbonyl, and methine carbon at the chiral center. This observed trend aligns with the interaction mechanism previously reported for the same chiral auxiliary. The configurational correlation can be effectively exploited under conditions of high dilution or, significantly, under sub-stoichiometric conditions.

## 1. Introduction

Determining the absolute configuration of chiral compounds is a critical step in chiral analysis and plays a key role in various chemical research areas, particularly in the synthesis and characterization of biologically relevant molecules. This task can be accomplished using different approaches, including optical spectroscopies such as circular dichroism [1,2,3,4], X-ray crystallography [4,5], mass spectrometry [4,6,7], or chromatographic techniques [4,8,9].

Nuclear magnetic resonance (NMR) spectroscopy has often been favored in chiral analysis due to its effectiveness, reliability, and versatility [10,11,12,13,14,15,16]. Specifically, chiral derivatizing agents (CDAs) have played a crucial role in NMR-based configurational assignments [12,13,14,15,16]. CDAs are enantiomerically pure chiral reagents that are employed to derivatize enantiomeric substrates to form their diastereomeric derivatives distinguishable in solution. The comparison of the chemical shifts of the two diastereomers can be correlated to their stereochemistry. Two approaches can be exploited depending on the availability of both enantiomers of the CDA or not. When both enantiomers of the CDA are available, two diastereomeric derivatives can be formed starting from each enantiomer of the substrate. In this case, the assignment of the absolute configuration of a single enantiomer can be achieved by comparing the sign of the chemical shift differences in selected protons in the two diastereomeric derivatives. If, instead, only one CDA enantiomer is available then the two enantiomers of the chiral substrate under investigation are mandatory, and their diastereomeric derivatives must be compared. Mosher’s acid (α-methoxy-α-trifluoromethylphenylacetic acid), which is commercially available in both enantiomeric forms, represents a milestone in the area of CDAs, and its applications have dominated the literature of configurational assignment by NMR since 1968 [17].

Chiral solvating agents (CSAs) have been used as well for such purposes [14], but their applications are strongly limited. Given that CSAs interact with the chiral substrates via labile non-covalent interactions, such as hydrogen bonding, dipole–dipole interaction, or π-π stacking, minor conformational preferences are found for the two CSA–substrate diastereomeric derivatives compared to covalently formed CDA–substrate diastereomers. Such enhanced conformational flexibility heavily impacts chemical shift differentiation of the two diastereomeric derivatives and, mainly, their interpretation for building reliable interaction models. On the other hand, the use of CSAs should be strongly encouraged for the practicality of their use, which simply requires mixing the CSA and the chiral substrate directly into the NMR tube in the suitable solvent. Moreover, the dependence of the chemical shift on several experimental parameters (like concentration, CSA-to-substrate molar ratio, temperature, and solvent) can be exploited to optimize the enantiodifferentiation. Also taking into account that every method for the assignment of the absolute configuration typically goes hand in hand with the determination of the enantiomeric purity, a further advantage related to the use of CSAs over CDAs is that the enantiomeric purity of the former does not undermine their applicability, as in the case of CDAs. Using CSAs for configurational assignment mirrors the established approaches utilized for CDAs, hinging on the availability of CSA enantiomers, be it one or both. Over the years, selected CSAs have been exploited for the analysis of chiral substrates such as trisubstituted allenes [18], natural products [19], hydroxy acids [20], and β-amino acids [21]. In recent decades, chiral-oriented media proved to be efficient tools for the chiral analysis of substrates lacking polar functional groups, which are instead fundamental for chiral discrimination by the majority of proposed CSAs [22,23].

A few years ago, Song demonstrated the efficacy of a new chiral sensor for *N*-protected α-amino acids based on a bis-thiourea CSA obtained by reacting 1,2-diphenylethane-1,2-diamine with 3,5-bis(trifluoromethyl)phenyl isothiocyanate [24]. In this case, a well-defined and reproducible correlation was found between the absolute configuration of the phthaloyl amino acid derivatives and the complexation shifts (Δδ = δ_mixture_ − δ_free_, where δ_mixture_ and δ_free_ are the chemical shifts of a given proton of the substrate in the presence and in the absence of the CSA, respectively) of their α-proton in the presence of the two enantiomers of the CSA [25].

**Figure 1:**
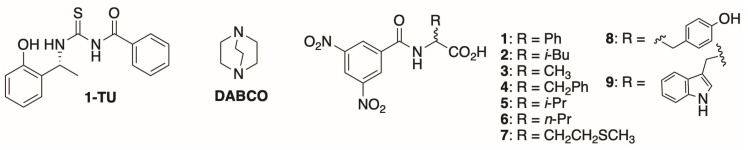
Structures of **1-TU**, DABCO, and *N*-DNB amino acids **1**–**9**.

Our recent contribution [26] to the development of new thiourea CSAs focused on the unexplored chiral platform 2-[(1*R*)-1-aminoethyl]phenol, which reacted with benzoyl isothiocyanate to prepare the thiourea derivative **1-TU** (Figure 1). **1-TU** was tested for its efficacy in the NMR differentiation of the enantiomers of *N*-3,5-dinitrobenzoyl (*N*-DNB) amino acid derivatives having free carboxyl functions. The outcomes were encouraging, as evidenced by the achievement of remarkably high enantiomeric differentiation in the NMR spectra [26]. The co-presence of a third achiral additive, the strong base 1,4-diazabicyclo[2.2.2]octane (DABCO, Figure 1), was crucial in order to improve the scarce solubility of amino acid derivatives, but it also cooperated with the CSA acting as a bridge in the stabilization of the diastereomeric solvates. A pivotal feature of our developed CSA was the 2-hydroxyphenyl group, which served a dual role: a strong hydrogen-bond donor group and an electron-donating aromatic moiety. For these reasons, this group played a fundamental role in promoting the hydrogen-bond network and in effectively attracting the 3,5-dinitrobenzoyl moiety of the amino acid derivative [26].

Having established the efficacy of 2-[(1*R*)-1-aminoethyl]phenol-based **1-TU** as a proficient CSA for determining the enantiomeric purity of mixtures of *N*-DNB amino acids, the current investigation delves into the unexploited potential of **1-TU** as a chiral sensor for the NMR determination of the absolute configuration for this class of compounds. The primary objective is to make a further contribution to the underexplored applications of CSAs in configurational assignment, thereby advancing the development of robust methods in this domain. In the pursuit of this objective, the versatility of **1-TU** towards *N*-DNB amino acids with the free carboxylic group was tested by expanding the repertoire of amino acid derivatives under consideration (**1**–**9**, Figure 1) with respect to the previous investigation [26]. The aim of this study was to affirm the reliability of the correlation between the ^1^H/^13^C NMR chemical shifts and the absolute configuration for all the substrates subjected to examination. The validity of the configurational assignment was explored by investigating several experimental conditions (concentration and CSA/substrate molar ratio) to assess the reliability of chemical shift/absolute configuration correlation.

## 2. Results and Discussions

**Scheme 1:**
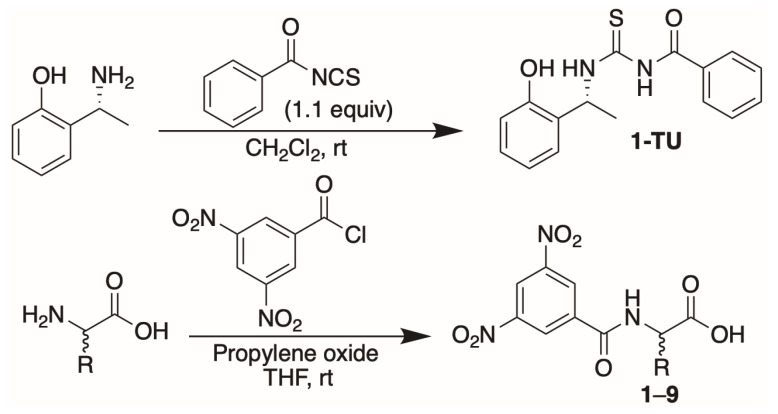
Synthesis of **1-TU** and amino acid derivatives **1**–**9**.

Derivatives **6**–**9** (Figure 1) were obtained by following the same experimental procedure already reported [26], involving the reaction between the amino acid and 3,5-dinitrobenzoyl chloride in the presence of propylene oxide (Scheme 1). The mono-thiourea **1-TU** (Scheme 1) was here exploited for the analysis of enantiomerically enriched mixtures of all *N*-DNB amino acids **1**–**9** (Figure 1).

Considering that only one enantiomer of **1-TU** was available, the configurational assignment procedure involved the analysis of ternary mixtures of CSA/chiral substrate/DABCO in deuterated chloroform (CDCl_3_), containing enantiomerically enriched mixtures of amino acid derivatives. Such experimental conditions, as previously reported [26], proved to be the most effective for the occurrence of the enantiodiscrimination processes.

Therefore, for ^1^H and ^13^C NMR enantiodiscrimination experiments, solutions under investigation consisted of 15 mM *N*-DNB amino acid, enriched in the (*S*)-enantiomer for **1**–**7** and in the (*R*)-enantiomer for **8** and **9**, along with two equivalents of CSA and one equivalent of DABCO. While these experimental conditions proved effective for most derivatives, complete solubilization of derivatives **8** and **9** was not achieved. Therefore, the concentration of these substrates was halved to 7.5 mM, while the concentration of CSA and DABCO was kept constant, resulting in a final CSA/substrate/DABCO molar ratio equal to 4:1:2. Despite the different experimental conditions adopted, the short-term stability of tryptophan derivative **9** with respect to the ^13^C acquisition time, notably longer than the proton one, precluded the recording of the carbon spectrum for the corresponding mixture.

**Figure 2:**
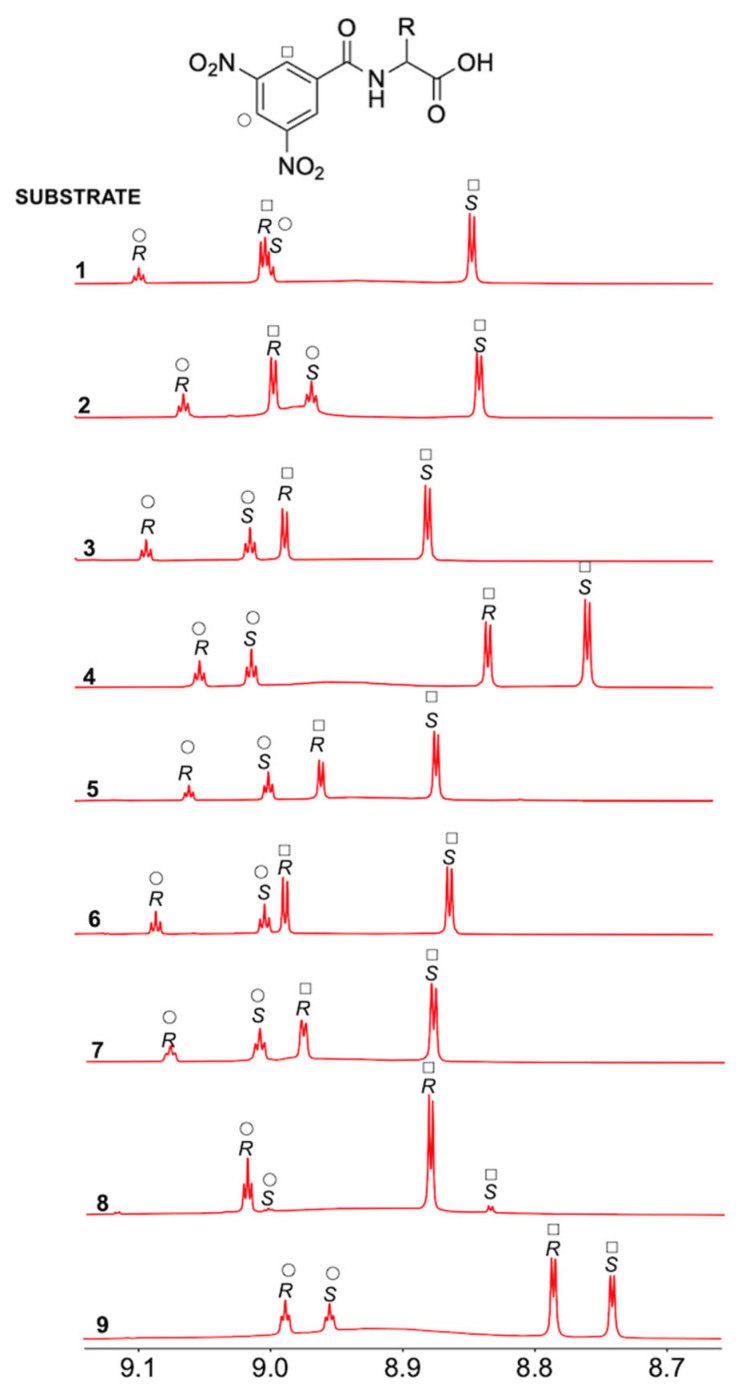
^1^H NMR (600 MHz, CDCl_3_, 25 °C) spectral regions corresponding to *N*-DNB protons of (*S*)-enantiomerically enriched mixtures of substrates **1**–**7** (15 mM) in the presence of 2 equiv of **1-TU** and 1 equiv of DABCO and of (*R*)-enantiomerically enriched mixtures of substrates **8** and **9** (7.5 mM) in the presence of 4 equiv of **1-TU** and 2 equiv of DABCO.

Table 1 collects proton nonequivalence data (ΔΔδ = |Δδ*^R^* − Δδ*^S^*| = |δ*^R^* − δ*^S^*|, where Δδ*^R^* = δ*^R^*_mixture_ − δ_free_ and Δδ*^S^* = δ*^S^*_mixture_ − δ_free_ are the complexation shifts) for substrates **1**–**9**, and Figure 2 highlights the spectral region where the *ortho* and *para* protons of the dinitrobenzoyl moiety resonate, revealing baseline differentiation for nearly all substrates. Even in the case of compound **1**, where a partial superimposition of signals was found (Figure 2), the relative positions of signals from the two enantiomers could be conclusively determined. Indeed, even though a clear correlation between the extent of nonequivalence and the structural features of the amino acid derivatives cannot be deduced, for all of them, the *ortho* and *para* protons of the 3,5-dinitrophenyl moiety in their (*R*)-enantiomers generate resonances that are shifted to higher frequencies compared to those of the (*S*)-enantiomers.

Encouraged by the consistent correlation between the absolute configuration and the relative positions of proton signals belonging to DNB moiety, an exploration of additional probes for configurational assignment was undertaken. This involved analyzing the methine protons for those systems where enantiodiscrimination was significant (**1**, **3**, **4**, **6**–**9**). For the CH nuclei, NMR techniques targeting spectral simplification by removing scalar coupling, such as pure shift experiments [27], can be particularly useful to improve the spectral resolution, thus overcoming the issues related to more complex multiplicities, especially in cases of low enantiodiscrimination.

The ^1^H NMR spectral regions of methine proton, along with the corresponding pure shift spectra for substrates **1**, **3**, **4**, and **6**–**9** are reported in Figure S1 in Supplementary Materials. An opposite sense of nonequivalence for the CH was found with respect to the DNB protons (Table 2, Figure S1, Supplementary Materials). As a matter of fact, the methine resonance of all the (*S*)-enantiomers were high-frequency shifted with respect to (*R*)-enantiomers.

In pursuit of developing an efficient method for configurational assignment, beyond the foundational step of identifying a consistent correlation between signal position and absolute configuration, it is crucial to define the limited experimental conditions (concentration and molar ratio) conducive to the successful realization of the assignment itself. This entails establishing the boundaries within which the signals of the two enantiomers remain distinguishable, surpassing the optimized conditions assessed so far. Naturally, these limits are less stringent than those required for enantiomeric purity determination, where the imperative is to obtain enantiomeric signals that can be integrated with precision. The configurational assignment is, in fact, viable as long as the signals of the two enantiomers do not entirely overlap.

**Figure 3:**
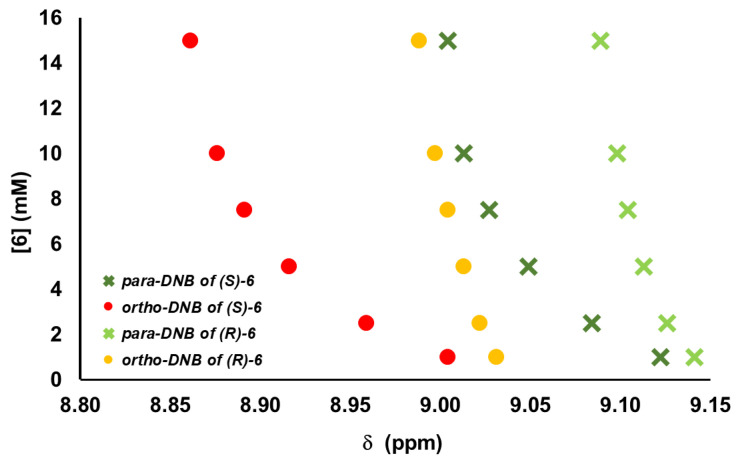
^1^H NMR (600 MHz, CDCl_3_, 25 °C) chemical shifts (ppm) of (*S*)-*ortho* (●), (*R*)-*ortho* (●), (*S*)-*para* (**×**), and (*R*)-*para* (**×**) protons in progressively diluted (*S*)-enantiomerically enriched mixtures of **6** in the presence of 2 equiv of **1-TU** and 1 equiv of DABCO.

**Figure 4:**
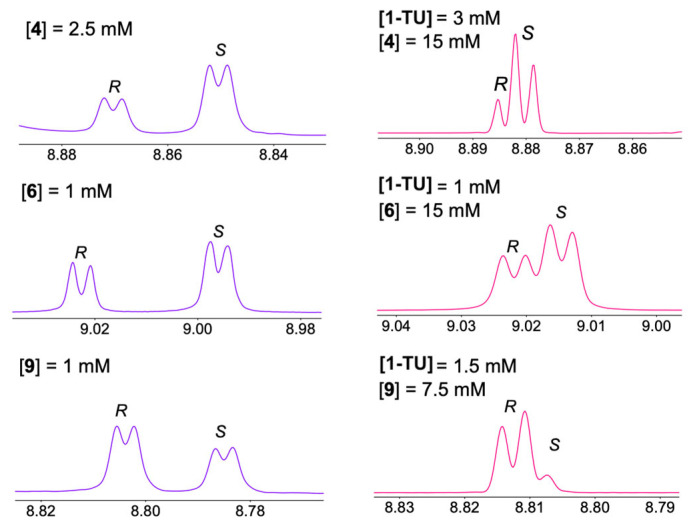
Left: ^1^H NMR (600 MHz, CDCl_3_, 25 °C) spectral regions corresponding to *ortho*-protons of (*S*)-enantiomerically enriched mixtures of **4** (2.5 mM) and **6** (1 mM) in the presence of 2 equiv of **1-TU** and 1 equiv of DABCO, and of (*R*)-enantiomerically enriched mixture of **9** (1 mM) in the presence of 4 equiv of **1-TU** and 2 equiv of DABCO. Right: ^1^H NMR (600 MHz, CDCl_3_, 25 °C) spectral regions corresponding to *ortho*-protons of (*S*)-enantiomerically enriched mixtures of **4** and **6** (15 mM) in the presence of **1-TU** (3 and 1 mM, respectively) and DABCO (15 mM) and of (*R*)-enantiomerically enriched mixture of **9** (7.5 mM) in the presence of **1-TU** (1.5 mM) and DABCO (15 mM).

To address this aspect, we focused on the nuclei of enantiomeric substrates exhibiting the highest sensitivity and optimal responsiveness to enantiomeric differentiation in the presence of **1-TU**, specifically, the *ortho* and *para* protons of the 3,5-dinitrobenzoyl moiety. Therefore, for selected amino acid derivatives, progressive dilution experiments were conducted by reducing the substrate concentration from 15 mM to 1 mM in mixtures **1-TU**/substrate/DABCO 2:1:1. Figure 3 illustrates the dependence of enantiomeric differentiation on total concentration (Tables S1 and S2 in Supplementary Materials) for compound **6**, which displayed significant nonequivalences at the maximum 15 mM concentration (Table 1). For this substrate, well-distinguishable signals were still detected at the lowest 1 mM concentration (Figure 3 and Figure 4). Considering derivative **4**, with the lowest nonequivalences at 15 mM (Table 1), the resonances of enantiomers remained distinguishable at 2.5 mM (Figure 4). Even for the less soluble tryptophan derivative **9**, analyzed starting from 7.5 mM, the two enantiomeric signals were distinguished down to 1 mM (Figure 4).

Furthermore, given the overarching goal of detecting enantiomeric signals regardless of their magnitude of differentiation, it is intriguing to explore sub-stoichiometric conditions where the CSA is in defect. This approach aims to minimize both CSA consumption and potential interference from CSA signals. Notably, highly stringent sub-stochiometric conditions for compound **6** could be explored, reaching substrate/DABCO/CSA ratios of 15:15:1. The limit conditions of concentration and substrate/CSA molar ratio were also assessed for compounds with lower nonequivalences, such as **4** and **9**. The results obtained for the aforementioned substrates **4**, **6**, and **9** are shown in Figure 4. In particular, at the concentration of 15 mM, phenylalanine derivative **4** allowed to attain distinguishable enantiomeric signals till to a substrate/CSA molar ratio equal to 5 to 1. Distinguishable signals of enantiomers of less soluble tryptophan derivative **9** at the same sub-stoichiometric 5-to-1 substrate/CSA molar ratio could be attained in more diluted conditions (substrate concentration of 7.5 mM).

In conclusion, it can be asserted that the method is applicable even under highly diluted conditions (2.5–1 mM) or sub-stochiometric ratios, with the substrate present in excess from five- to fifteen-fold compared to the CSA.

To bolster the ^1^H NMR investigation on enantiomerically enriched samples **1**–**9,** a complementary ^13^C NMR analysis was conducted. The observation of ^13^C nuclei has often been underestimated in applications related to chiral analysis. This is primarily attributed to the lower isotopic abundance and the lower gyromagnetic ratio of ^13^C nuclei compared to ^1^H ones, resulting in inherently lower sensitivity and, consequently, longer times of acquisition of spectra. Another challenge lies in the longer relaxation times, particularly for quaternary carbons, making the quantitative response less reliable. The increasing availability of high-field spectrometers, however, has rendered low-sensitivity concerns less significant and highlighted the strengths of ^13^C NMR spectroscopy. Specifically, the significantly narrower line widths of ^13^C signals and the greater spectral dispersion have become more pronounced with high-field instruments. In particular, the latter characteristic makes ^13^C NMR spectroscopy particularly compelling in configurational assignment issues. In such cases, the paramount goal is to achieve an unequivocal observation of the relative position of NMR signals from the two enantiomers, where the enhanced spectral dispersion plays a crucial role.

Therefore, the same samples utilized for ^1^H NMR analysis, i.e., 2:1:1 CSA/substrate (15 mM)/DABCO for **1**–**7** and 4:1:2 CSA/substrate (7.5 mM)/DABCO for **8** were subjected to ^13^C NMR analysis. As already said, a mixture of **9** could not be analyzed via ^13^C NMR due to its short-term stability. Remarkably high ^13^C nonequivalence data were measured for substrates **1**–**7** (Table 3) for the quaternary carbons of the *N*-DNB moiety (*C*-NO_2_, 0.138–0.360 ppm), for the carbonyl of the amide (0.183–0.403 ppm) and the carboxyl groups (0.266–0.436 ppm), and for the methine carbon at the chiral center (0.102–0.267 ppm). Notably, the quaternary carbons exhibited the highest degrees of nonequivalences (Table 3). For substrate **8**, lower nonequivalence values were measured primarily confined to the carbons of the DNB moiety (Table 3).

By comparing ^13^C NMR data acquired under the current experimental conditions to those reported in our prior work [26], where equimolar mixtures of CSA/substrate (30 mM)/DABCO were analyzed for substrates **1** and **2**, a notable observation arises. It becomes evident that the nonequivalences measured for the quaternary carbons are more influenced by the molar ratio rather than the total concentration, exhibiting a significant increase under the conditions selected for this study. For instance, nonequivalences of 0.405 ppm and 0.436 ppm were measured for the carboxylic carbons of the derivatives **1** and **2**, respectively, in comparison to the values of 0.313 ppm and 0.328 ppm previously obtained [26].

**Figure 5:**
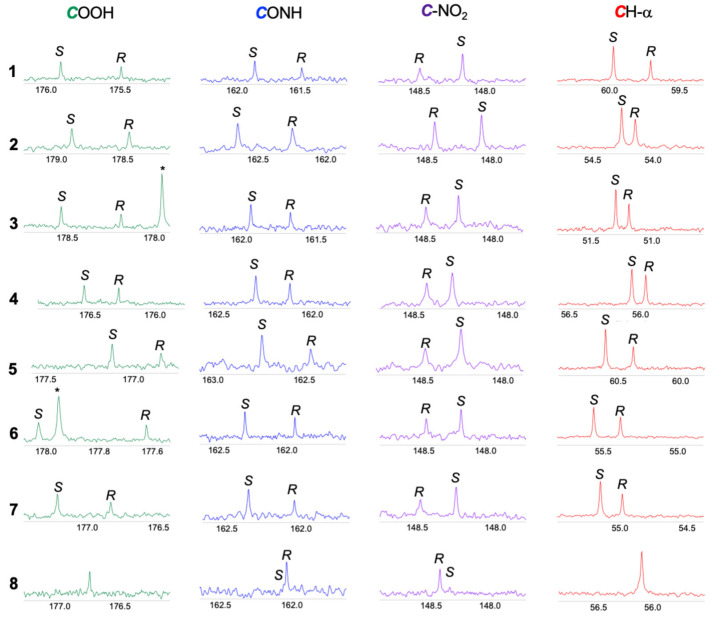
^13^C{^1^H} NMR (150 MHz, CDCl_3_, 25 °C) spectral regions corresponding to carboxylic (green), amidic (blue), *C*-NO_2_ (purple) and methine (red) carbons of (*S*)-enantiomerically enriched mixtures of substrates **1**–**7** (15 mM) in the presence of 2 equiv of **1-TU** and 1 equiv of DABCO and of (*R*)-enantiomerically enriched mixture of **8** (7.5 mM) in the presence of 4 equiv of **1-TU** and 2 equiv of DABCO. * indicates **1-TU** resonance.

In Figure 5 the ^13^C NMR spectral regions corresponding to the carbons reported in Table 3 are shown for substrates **1**–**8**. Moving to the analysis of the relative position of the two enantiomers, a reproducible correlation between the sense of nonequivalence and the absolute configuration was obtained for the ^13^C NMR signals of quaternary *C*-NO_2_, carboxylic, amidic and CH-α carbons of **1**–**8** (Figure 5).

As shown in Figure 5 and in Table 4, the carboxylic, amidic, and CH-α carbons of the (*R*)-enantiomers were low-frequency shifted with respect to the (*S*)-enantiomers; carbons directly bonded to nitro groups, on the contrary, were high-frequency shifted in the diastereomeric derivatives of the (*R*)-substrates.

**Figure 6:**
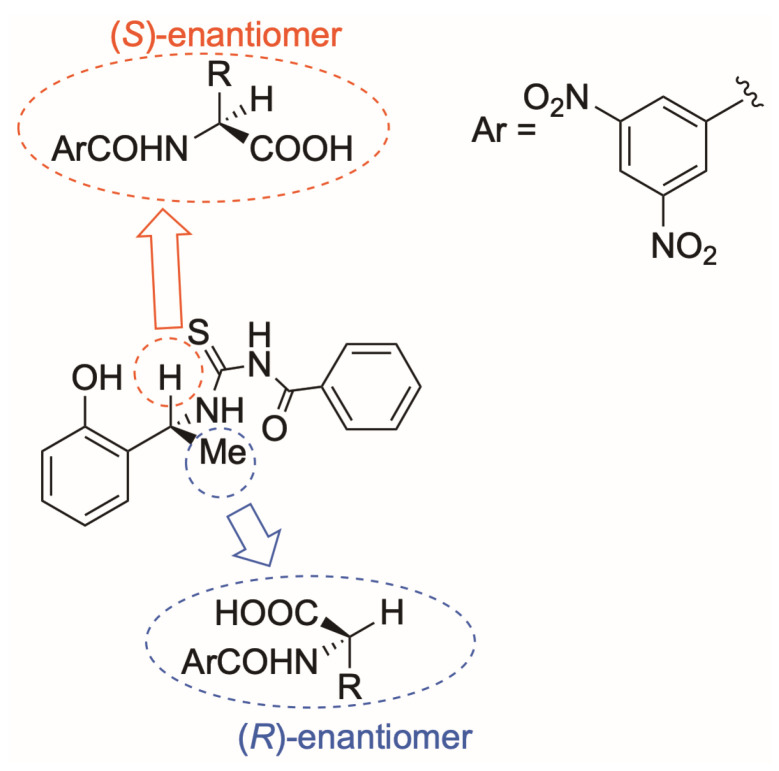
Schematic representation of the interaction between **1-TU** and (*R*)-/(*S*)-amino acid derivative.

Attempting to correlate the observations made so far regarding the relationship between the sense of nonequivalence and the absolute configuration, we can refer to the interaction model of **1-TU** with the two enantiomers of the amino acid derivatives, previously proposed [26] and schematically represented here in Figure 6. Firstly, what governs the interaction mechanism is the need to keep the carboxyl group always in spatial proximity to the CSA. This interaction is mediated by DABCO, which deprotonates the carboxyl group of the substrate, making the carboxylate more suitable to interact with the acidic NH groups of the thiourea moiety and, importantly, with the acidic phenolic hydroxyl as well. The additional stabilizing interaction involves the attraction between the π-basic 2-hydroxyphenyl group of **1-TU** and the π-acid 3,5-dinitrobenzoyl group of the amino acid derivative. This interaction plays a pivotal role in producing the observed shielding effects (Table 2) for nearly all the *ortho* and *para* protons of the DNB group for both enantiomers. Deshielding effects (Table 2) detected for the methine protons of enantiomeric substrate pairs can be ascribed to the benzoyl moiety. Previously reported NOE effects [26] have convincingly demonstrated that the (*S*)-enantiomer engages with the **1-TU** face, offering minor steric hindrance, specifically the one encompassing the methine proton linked to the chiral center of **1-TU** itself. On the other hand, the other enantiomer, likely to preserve the pool of aforementioned attractive interactions, interacts with the face of **1-TU** housing its methyl group. Consequently, both enantiomers undergo qualitatively similar effects, namely the shielding of their DNB protons and the deshielding effects of the methine proton. However, these effects are subdued for the (*R*)-enantiomer, whose interaction with **1-TU** is weakened by steric repulsion exerted by the methyl group of **1-TU** itself. This result aligns with the previously measured [26] lower association constant for the (*R*)-substrate/**1-TU** complex compared to the (*S*)-substrate/**1-TU** complex.

The complexation shifts observed for the carbon resonances (Table 4) were in agreement with the data observed for the ^1^H NMR nuclei (Table 2). As a matter of fact, the same trend in the relative positions of the *ortho* and *para* protons was found for the quaternary carbons belonging to the 3,5-dinitrophenyl moiety, as well as for the ^1^H and ^13^C nuclei of the stereocenters (Figure 2 and Figure 5, Table 2 and Table 4, and Figure S1 in Supplementary Materials).

## 3. Materials and Methods

### 3.1. Materials

All commercially available substrates, reagents, and solvents were purchased from Sigma-Aldrich (Darmstadt, Germany) and used without further purification. Tetrahydrofuran (THF) was distilled from sodium. Deuterated chloroform (CDCl_3_) was purchased from Deutero GmbH (Kastellaun, Germany).

### 3.2. Methods

^1^H and ^13^C{^1^H} NMR measurements were carried out in CDCl_3_ on a Varian INOVA600 (Varian, Pao Alto, CA, USA) spectrometer equipped with a 5 mm probe operating at 600 MHz and 150 MHz for ^1^H and ^13^C nuclei, respectively. ^1^H and ^13^C chemical shifts were referred to tetramethylsilane (TMS) as the secondary reference standard, and the temperature was controlled (25 ± 0.1 °C). The ^1^H NMR spectra were recorded by using the minimum spectral width required, a relaxion delay of 5 s, 1.8 s of acquisition time, a gain of 20, and 32 scans; the π/2 pulse was optimized for each sample. The pure shift spectra were recorded by using the PS1D (Zangger–Sterk, Southampton, UK) pulse sequence, a relaxion delay of 2 s, 0.4 s of acquisition time, a gain of 20, and 32 scans; a slice selection bandwidth of 120 Hz, and a τ-delay of 14.9 ms were selected. The ^13^C{^1^H} NMR spectra were recorded using a relaxation delay of 1 s, 0.9 s of acquisition time, a gain of 30, and 14,400 scans.

### 3.3. Synthesis of N-3,5-Dinitrobenzoyl Amino Acids ***6***–***9***

Derivatives **6**–**9** were synthesized according to the literature [26]; the characterization data are shown below. NMR numbering schemes are reported in the ^1^H and ^13^C NMR spectra in Supplementary Materials (Figures S2–S9).

**6**: white solid; 76% yield. ^1^H NMR (600 MHz, 15 mM, CDCl_3_, 25 °C), δ (ppm): 9.13 (t, ^4^*J* = 2.1 Hz, ^1^H, H_1_), 9.06 (d, ^4^*J* = 2.1 Hz, 2H, H_2_), 7.75 (d, ^3^*J* = 6.6 Hz, 1H, H_3_), 4.69 (ddd, ^3^*J* = 6.6 Hz, ^3^*J* = 4.9 Hz, ^3^*J* = 4.9 Hz, 1H, H_4_), 1.98 (m, 1H, H_5_), 1.81 (m, 1H, H_5’_), 1.42 (m, 1H, H_6_), 1.37 (m, 1H, H_6’_), 1.24 (t, ^3^*J* = 7.4 Hz, 3H, H_7_). ^13^C NMR (150 MHz, 15 mM, CDCl_3_, 25 °C), δ (ppm): 177.1 (C_7_), 161.9 (C_5_), 148.6 (C_2_), 138.2 (C_4_), 127.4 (C_3_), 120.9 (C_1_), 54.5 (C_6_), 35.0 (C_8_), 18.8 (C_9_), 13.9 (C_10_).

**7**: white solid; 75% yield. ^1^H NMR (600 MHz, 15 mM, CDCl_3_, 25 °C), δ (ppm): 9.08 (t, ^4^*J* = 2.0 Hz, 1H, H_1_), 8.96 (d, ^4^*J* = 2.0, 2H, H_2_), 8.59 (d, ^3^*J* = 6.8 Hz, 1H, H_3_), 4.62 (ddd, ^3^*J* = 6.8 Hz, ^3^*J* = 4.6 Hz, ^3^*J* = 4.6 Hz, 1H, H_4_), 2.59 (m, 2H, H_5/5’_), 2.35 (m, 1H, H_6_), 2.12 (m, 1H, H_6’_), 2.09 (s, 3H, H_7_). ^13^C NMR (150 MHz, 15 mM, CDCl_3_, 25 °C), δ (ppm): 176.9 (C_7_), 161.6 (C_5_), 148.5 (C_2_), 138.1 (C_4_), 127.3 (C_3_), 120.7 (C_1_), 54.7 (C_6_), 32.5 (C_8_), 30.8 (C_9_), 15.6 (C_10_).

**8**: yellow solid; 53% yield. ^1^H NMR (600 MHz, 15 mM, CDCl_3_, 25 °C), δ (ppm): 9.08 (t, ^4^*J* = 2.0 Hz, 1H, H_1_), 8.88 (d, ^4^*J* = 2.0 Hz, 2H, H_2_), 7.36 (d, ^3^*J* = 6.1 Hz, 1H, H_3_), 6. 97 (d, ^3^*J* = 6.7 Hz, 2H, H_6_), 6.54 (d, ^3^*J* = 6.7 Hz, 2H, H_7_), 4.77 (ddd, ^3^*J* = 6.1 Hz, ^3^*J* = 4.6 Hz, ^3^*J* = 4.6 Hz, 1H, H_4_), 3.25 (dd, ^2^*J* = 13.9 Hz, ^3^*J* = 4.6 Hz, 1H, H_5_), 3.22 (dd, ^2^*J* = 13.9 Hz, ^3^*J* = 4.6 Hz, 1H, H_5’_). ^13^C NMR (150 MHz, 15 mM, CDCl_3_, 25 °C), δ (ppm): 176.4 (C_7_), 161.8 (C_5_), 155.9 (C_12_), 148.5 (C_2_), 138.7 (C_4_), 130.6 (C_10_), 127.9 (C_9_), 127.3 (C_3_), 120.7 (C_1_), 115.3 (C_11_), 55.9 (C_6_), 36.7 (C_8_).

**9**: orange crystalline solid; 51% yield. ^1^H NMR (600 MHz, 15 mM, CDCl_3_, 25 °C), δ (ppm): 9.04 (s, 1H, H_1_), 8.79 (s, 2H, H_2_), 8.58 (s, 1H, H_7_), 7.70 (bs, 1H, H_3_), 7.64 (d, ^3^*J* = 7.0 Hz, 1H, H_11_), 7.29 (d, ^3^*J* = 7.0 Hz, 1H, H_8_), 7.09 (t, ^3^*J* = 7.0 Hz, 1H, H_9_), 7.00 (s, 1H, H_6_), 6.96 (t, ^3^*J* = 7.0 Hz, 1H, H_10_), 4.84 (m, 1H, H_4_), 3.56 (dd, ^2^*J* = 14.8 Hz, ^3^*J* = 4.8 Hz, 1H, H_5_), 3.50 (dd, ^2^*J* = 14.8 Hz, ^3^*J* = 4.8 Hz, 1H, H_5’_). ^13^C NMR (150 MHz, 15 mM, CDCl_3_, 25 °C), δ (ppm): 176.8 (C_7_), 161.8 (C_5_), 148.3 (C_2_), 138.5 (C_11_), 136.1 (C_4_), 128.2 (C_16_), 127.2 (C_3_), 122.5 (C_10_), 121.8 (C_13_), 120.5 (C_1_), 119.1 (C_14_), 119.0 (C_15_), 111.8 (C_9_), 111.2 (C_12_), 55.4 (C_6_), 27.4 (C_8_).

### 3.4. Preparation of Enantiomerically Enriched Mixtures for ^1^H and ^13^C NMR Experiments

Chiral solvating agent **1-TU** (30 mM), DABCO (15 mM with **1**–**7** and 30 mM with **8** and **9**), and (*R*)-/(*S*)-DNB amino acids (15 mM for **1**–**7** and 7.5 mM for **8** and **9**) were mixed in CDCl_3_ (0.6 mL). (*S*)-enantiomerically enriched samples of **1**–**7** were prepared with an enantiomeric excess of −24% (**1**), −19% (**2**), −22% (**3**), −14% (**4**), −36% (**5**), −24% (**6**), and −34% (**7**). (*R*)-enantiomerically enriched samples of **8** and **9** were prepared with an enantiomeric excess of +92% (**8**) and +13% (**9**).

## 4. Conclusions

The pursuit of analytical methods to determine the absolute configuration of amino acids or their derivatives is anything but a peripheral endeavor in the field of chiral analysis. Given their vital role in the construction of peptides and proteins, these compounds hold paramount importance as foundational components in living systems. Racemization processes, occurring in the dependence on pH conditions, can intricately alter the conformational dynamics of supramolecular derivatives and, consequently, have profound effects on the biological activity of proteins [28,29]. Thus, reliable analytical methodologies for establishing stereoisomeric purity and the absolute configuration of individual amino acid constituents are essential.

The development of stereoselective synthetic procedures for amino acid analogs is also intricately tied to the need for reliable and precise methods of chiral analysis [30,31,32,33].

Despite abundant potential, applications of CSAs in NMR determination of the absolute configuration of amino acids have been relatively scarce.

In this study, the thiourea derivative of 2-[(1*R*)-1-aminoethyl]phenol (**1-TU**) emerged as an effective tool for assigning the absolute configuration of 3,5-dinitrobenzoyl derivatives of amino acids with free carboxyl functions, in the presence of DABCO. The practicality of **1-TU** synthesis and of the derivatization procedure for amino acids, coupled with the fact that the 3,5-dinitrophenyl moiety generates resonances in a high-frequency spectral region unaffected by interference from CSA signals, makes the method particularly attractive.

A reproducible correlation between the relative position of resonances and the absolute configuration has been observed not only for ^1^H nuclei but also for several ^13^C nuclei of the amino acid derivatives. The phenolic and benzoyl moieties of the CSA induce shielding effects on the enantiomers, elucidating a precise sense of nonequivalence. Importantly, the fact that the correlation is maintained even under conditions of progressive dilution or sub-stoichiometric molar ratio (in defect of CSA) reassures about the validity of the method across a broad range of experimental conditions.

Therefore, the encouragement for the use of CSAs in determining the absolute configuration of amino acid derivatives, complementing their established role in enantiomeric purity determinations, is further emphasized.

## Figures and Tables

**Table 1:** ^1^H NMR nonequivalences (ΔΔδ, ppm, 600 MHz, CDCl_3_, 25 °C) for *ortho*-/*para*-DNB and CH-α protons of (*S*)-enantiomerically enriched mixtures of substrates **1**–**7** (15 mM) in the presence of 2 equiv of **1-TU** and 1 equiv of DABCO and of (*R*)-enantiomerically enriched mixtures of substrates **8** and **9** (7.5 mM) in the presence of 4 equiv of **1-TU** and 2 equiv of DABCO.

Sub	ΔΔδ		
	*ortho*-DNB	*para*-DNB	CH-α
**1** *	0.156	0.096	0.039
**2** *	0.132	0.082	0.004
**3** *	0.134	0.095	0.037
**4** *	0.034	0.016	0.026
**5** *	0.084	0.055	0.008
**6**	0.129	0.085	0.017
**7**	0.102	0.070	0.019
**8**	0.055	0.019	0.018
**9**	0.054	0.041	0.026

* Data from reference [26].

**Table 2:** ^1^H complexation shifts (Δδ = δ_mixture_ − δ_free_, ppm, 600 MHz, CDCl_3_, 25 °C) for ortho-/para-DNB and CH-α protons of (*S*)-enantiomerically enriched mixtures of substrates **1**–**7** (15 mM) in the presence of 2 equiv of **1-TU** and 1 equiv of DABCO and of (*R*)-enantiomerically enriched mixtures of substrates **8** and **9** (7.5 mM) in the presence of 4 equiv of **1-TU** and 2 equiv of DABCO.

Sub	Δδ*^R^*			Δδ*^S^*		
	*ortho*-DNB	*para*-DNB	CH-α	*ortho*-DNB	*para*-DNB	CH-α
**1**	−0.002	−0.016	0.057	−0.158	−0.112	0.096
**2**	0.041	−0.011	0.076	−0.091	−0.093	0.080
**3**	−0.003	−0.033	0.019	−0.137	−0.128	0.056
**4**	−0.051	−0.044	0.007	−0.085	−0.060	0.033
**5**	0.013	−0.045	0.062	−0.071	−0.100	0.070
**6**	−0.012	−0.036	−0.015	−0.141	−0.121	0.002
**7**	0.021	0.005	0.011	−0.081	−0.065	0.030
**8**	−0.008	−0.037	0.056	−0.063	−0.056	0.074
**9**	−0.032	−0.046	0.041	−0.086	−0.087	0.067

**Table 3:** ^13^C{^1^H} NMR nonequivalences (ΔΔδ, ppm, 150 MHz, CDCl_3_, 25 °C) for *C*-NO_2_, *C*ONH, *C*OOH and CH-α carbons of (*S*)-enantiomerically enriched mixtures of substrates **1**–**7** (15 mM) in the presence of 2 equiv of **1-TU** and 1 equiv of DABCO and of (*R*)-enantiomerically enriched mixture of **8** (7.5 mM) in the presence of 4 equiv of **1-TU** and 2 equiv of DABCO.

Sub	ΔΔδ			
	*C*OOH	*C*ONH	*C*-NO_2_	CH-α
**1**	0.405	0.377	0.328	0.267
**2**	0.436	0.403	0.360	0.115
**3**	0.345	0.269	0.241	0.107
**4**	0.266	0.183	0.138	0.102
**5**	0.275	0.266	0.222	0.222
**6**	0.405	0.367	0.284	0.184
**7**	0.374	0.314	0.230	0.170
**8**	0	0.055	0.023	0

**Table 4:** ^13^C complexation shifts (Δδ = Δδ_mixture_ − δ_free_, ppm, 150 MHz, CDCl_3_, 25 °C) for *C*-NO_2_, *C*ONH, *C*OOH and CH-α carbons of (*S*)-enantiomerically enriched mixtures of substrates **1**–**7** (15 mM) in the presence of 2 equiv of **1-TU** and 1 equiv of DABCO and of (*R*)-enantiomerically enriched mixtures of **8** (7.5 mM) in the presence of 4 equiv of **1-TU** and 2 equiv of DABCO.

Sub	Δδ*^R^*				Δδ*^S^*			
	*C*OOH	*C*ONH	*C*-NO_2_	CH-α	*C*OOH	*C*ONH	*C*-NO_2_	CH-α
**1**	0.275	0.406	−0.004	0.509	0.680	0.783	−0.332	0.776
**2**	0.404	0.542	−0.068	0.512	0.840	0.945	−0.428	0.627
**3**	0.251	0.304	−0.012	0.305	0.596	0.573	−0.253	0.412
**4**	0.289	0.449	−0.085	0.189	0.555	0.632	−0.223	0.291
**5**	0.444	0.366	−0.082	0.460	0.719	0.632	−0.304	0.682
**6**	0.511	0.046	−0.108	0.856	0.916	0.413	−0.392	1.040
**7**	−0.069	0.399	0.015	0.257	0.305	0.713	−0.215	0.427
**8**	−0.458	−0.152	−0.109	0.046	−0.458	−0.207	−0.132	0.046

## Data Availability

Data are contained within the article or Supplementary Materials.

## Supplementary Materials

The following supporting information can be downloaded at https://www.mdpi.com/article/10.3390/molecules29061319/s1. Figure S1: ^1^H NMR (600 MHz, CDCl_3_, 25 °C) spectral regions (blue) and pure shift spectral regions (green) corresponding to CH-α protons of (*S*)-enantiomerically enriched mixtures of substrate **1**, **3, 4**, **6**, and **7** (15 mM) in the presence of 2 equiv of **1-TU** and 1 equiv of DABCO and of (*R*)-enantiomerically enriched mixture of **8** and **9** (7.5 mM) in the presence of 4 equiv of **1-TU** and 2 equiv of DABCO; Table S1: ^1^H NMR (600 MHz, CDCl_3_, 25 °C) chemical shifts (δ, ppm) measured for the DNB protons of (*S*)-**6** and (*R*)-**6** at different concentrations (mM) in the presence of 2 equiv of **1-TU** and 1 equiv of DABCO; Table S2: ^1^H NMR (600 MHz, CDCl_3_, 25 °C) chemical shifts (δ, ppm) measured for the DNB protons of (*S*)-**6** and (*R*)-**6** at different concentrations (mM) in the presence of 1 equiv of **1-TU** and 1 equiv of DABCO; Figure S2: ^1^H NMR (600 MHz, CDCl_3_, 25 °C) spectrum of **6** (15 mM) in the presence of 1 equiv of DABCO; Figure S3: ^13^C{^1^H} NMR (150 MHz, CDCl_3_, 25 °C) spectrum of **6** (15 mM) in the presence of 1 equiv of DABCO; Figure S4: ^1^H NMR (600 MHz, CDCl_3_, 25 °C) spectrum of **7** (15 mM) in the presence of 1 equiv of DABCO; Figure S5: ^13^C{^1^H} NMR (150 MHz, CDCl_3_, 25 °C) spectrum of **7** (15 mM) in the presence of 1 equiv of DABCO; Figure S6: ^1^H NMR (600 MHz, CDCl_3_, 25 °C) spectrum of **8** (15 mM) in the presence of 1 equiv of DABCO; Figure S7: ^13^C{^1^H} NMR (150 MHz, CDCl_3_, 25 °C) spectrum of **8** (15 mM) in the presence of 1 equiv of DABCO; Figure S8: ^1^H NMR (600 MHz, CDCl_3_, 25 °C) spectrum of **9** (15 mM) in the presence of 1 equiv of DABCO; Figure S9: ^13^C{^1^H} NMR (150 MHz, CDCl_3_, 25 °C) spectrum of **9** (15 mM) in the presence of 1 equiv of DABCO.
