# Supplementary material for: A Thiourea Derivative of 2-[(1R)-1-Aminoethyl]phenol as a Chiral Sensor for the Determination of the Absolute Configuration of N-3,5-Dinitrobenzoyl Derivatives of Amino Acids
Source: Molecules. 2024 Mar 15;29(6):1319. doi: 10.3390/molecules29061319 (PMC10974230; doi:10.3390/molecules29061319)
Supplement: Supplementary file 1 [file molecules-29-01319-s001.zip › molecules-2907095-supplementary.pdf]

A Thiourea Derivative of 2-[(1R)-1-Aminoethyl]phenol as a Chiral Sensor for the Determination of the Absolute Configuration of N-3,5-Dinitrobenzoyl Derivatives of Amino Acids

Federica Aiello 1, Alessandra Recchimurzo 2, Federica Balzano 2,*, Gloria Uccello Barretta 2,* and Federica Cefalì 2

1 Institute for Chemical and Physical Processes, National Research Council, Via G. Moruzzi 1, 56124 Pisa, Italy; federica.aiello@cnr.it

2 Department of Chemistry and Industrial Chemistry, University of Pisa, Via G. Moruzzi 13, 56124 Pisa, Italy; alessandra.recchimurzo@phd.unipi.it (A.R.); f.cefali@studenti.unipi.it (F.C.)

* Correspondence: federica.balzano@unipi.it (F.B.); gloria.uccello.barretta@unipi.it (G.U.B.)

Supplementary Materials

Table of contents

Figure S1. ^{1}H NMR (600 MHz, $CDCl_3$, 25 °C) spectral regions (blue) and Pure Shift spectral regions (green) corresponding to CH-α protons of (*S*)-enantiomerically enriched mixtures of substrate **1**, **3**, **4**, **6** and **7** (15mM) in the presence of 2 equiv of **1-TU** and 1 equiv of DABCO and of (*R*)-enantiomerically enriched mixture of **8** and **9** (7.5 mM) in the presence of 4 equiv of **1-TU** and 2 equiv of DABCO.

Table S1. ^{1}H NMR (600 MHz, $CDCl_3$, 25 °C) chemical shifts (δ, ppm) measured for the DNB protons of (*S*)-**6** and (*R*)-**6** at different concentrations (mM) in the presence of 2 equiv of **1-TU** and 1 equiv of DABCO.

Table S2. ^{1}H NMR (600 MHz, $CDCl_3$, 25 °C) chemical shifts (δ, ppm) measured for the DNB protons of (*S*)-**6** and (*R*)-**6** at different concentrations (mM) in the presence of 1 equiv of **1-TU** and 1 equiv of DABCO.

Figure S2. ^{1}H NMR (600 MHz, $CDCl_3$, 25 °C) spectrum of **6** (15 mM) in the presence of 1 equiv of DABCO.

Figure S3. ^{13}C{^{1}H} NMR (150 MHz, $CDCl_3$, 25 °C) spectrum of **6** (15 mM) in the presence of 1 equiv of DABCO.

Figure S4. ^{1}H NMR (600 MHz, $CDCl_3$, 25 °C) spectrum of **7** (15 mM) in the presence of 1 equiv of DABCO.

Figure S5. ^{13}C{^{1}H} NMR (150 MHz, $CDCl_3$, 25 °C) spectrum of **7** (15 mM) in the presence of 1 equiv of DABCO.

Figure S6. ^{1}H NMR (600 MHz, $CDCl_3$, 25 °C) spectrum of **8** (15 mM) in the presence of 1 equiv of DABCO.

Figure S7. ^{13}C{^{1}H} NMR (150 MHz, $CDCl_3$, 25 °C) spectrum of **8** (15 mM) in the presence of 1 equiv of DABCO.

Figure S8. ^{1}H NMR (600 MHz, $CDCl_3$, 25 °C) spectrum of **9** (15 mM) in the presence of 1 equiv of DABCO.

Figure S9. ^{13}C{^{1}H} NMR (150 MHz, 25 °C, $CDCl_3$) spectrum of **9** (15 mM) in the presence of 1 equiv of DABCO.

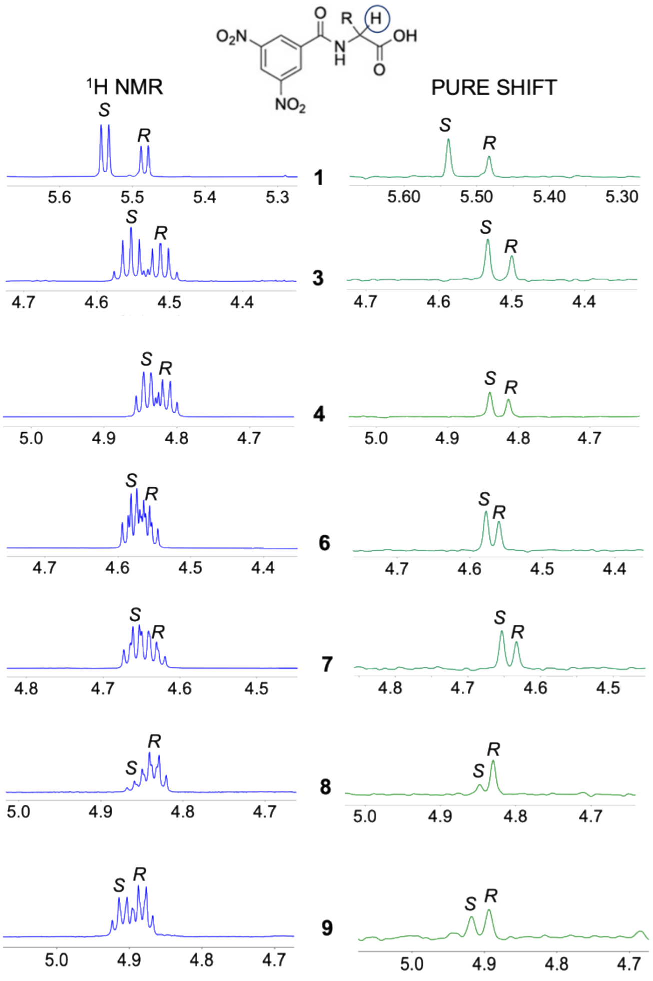

Figure S1. ^{1}H NMR (600 MHz, $CDCl_3$, 25 °C) spectral regions (blue) and Pure Shift spectral regions (green) corresponding to CH-α protons of (*S*)-enantiomerically enriched mixtures of substrate **1**, **3**, **4**, **6** and **7** (15mM) in the presence of 2 equiv of **1-TU** and 1 equiv of DABCO and of (*R*)-enantiomerically enriched mixture of **8** and **9** (7.5 mM) in the presence of 4 equiv of **1-TU** and 2 equiv of DABCO.

Table S1. ^{1}H NMR (600 MHz, $CDCl_3$, 25 °C) chemical shifts (δ, ppm) measured for the DNB protons of (*S*)-**6** and (*R*)-**6** at different concentrations (mM) in the presence of 2 equiv of **1-TU** and 1 equiv of DABCO.

	***para*-DNB**	***para*-DNB**	***ortho*-DNB**	***ortho*-DNB**
**C (mM)**	**δ_R (ppm)**	**δ_S (ppm)**	**δ_R (ppm)**	**δ_S (ppm)**
15	9.089	9.004	8.988	8.861
10	9.098	9.013	8.997	8.876
7.5	9.104	9.027	9.004	8.891
5	9.113	9.049	9.013	8.916
2.5	9.126	9.084	9.022	8.959
1	9.141	9.122	9.031	9.004

Table S2. ^{1}H NMR (600 MHz, $CDCl_3$, 25 °C) chemical shifts (δ, ppm) measured for the DNB protons of (*S*)-**6** and (*R*)-**6** at different concentrations (mM) in the presence of 1 equiv of **1-TU** and 1 equiv of DABCO.

C (mM)	*para*-DNB δR (ppm)	*para*-DNB δS (ppm)	*ortho*-DNB δR (ppm)	*ortho*-DNB δS (ppm)
15	9.105	9.052	9.028	8.944
10	9.115	9.075	9.034	8.973
7.5	9.126	9.102	9.038	9.003
5	9.135	9.122	9.040	9.021
2.5	9.149	9.145	9.027	9.024
1	9.105	9.052	9.028	8.944

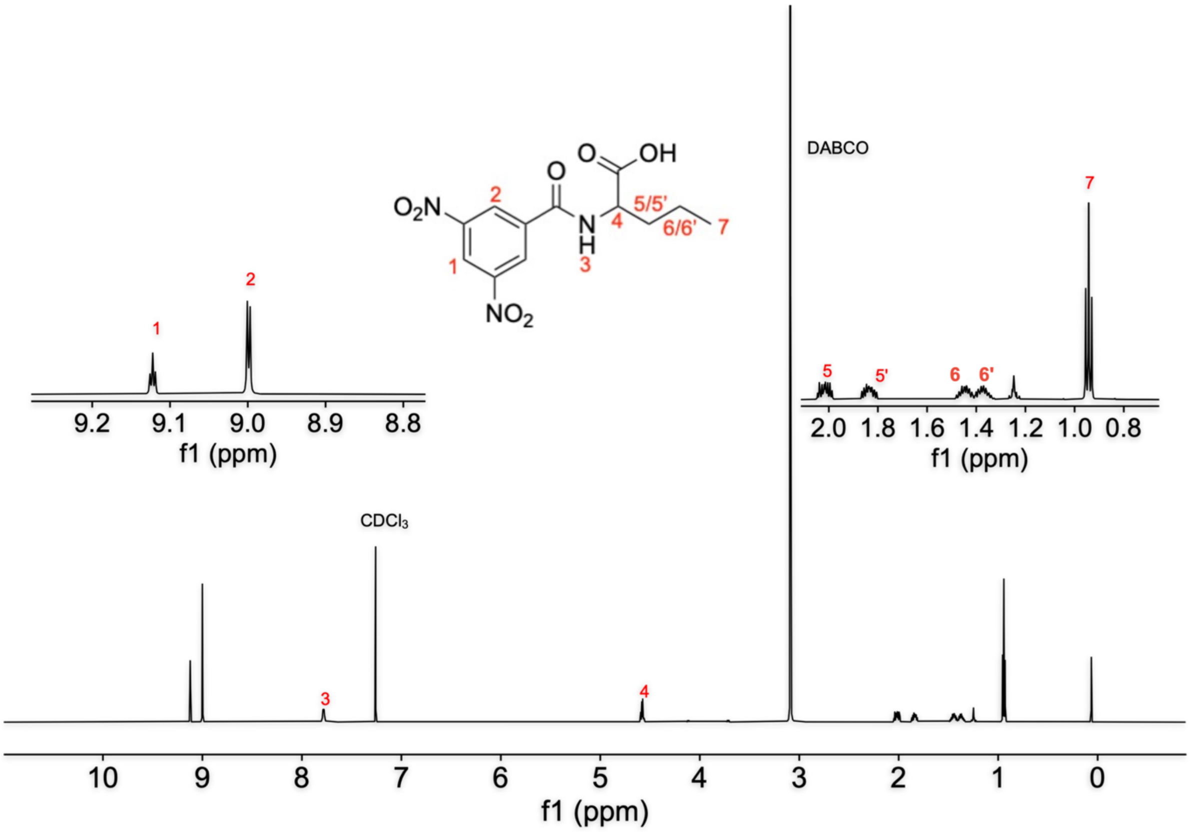

Figure S2. ^{1}H NMR (600 MHz, $CDCl_3$, 25 °C) spectrum of **6** (15 mM) in the presence of 1 equiv of DABCO.

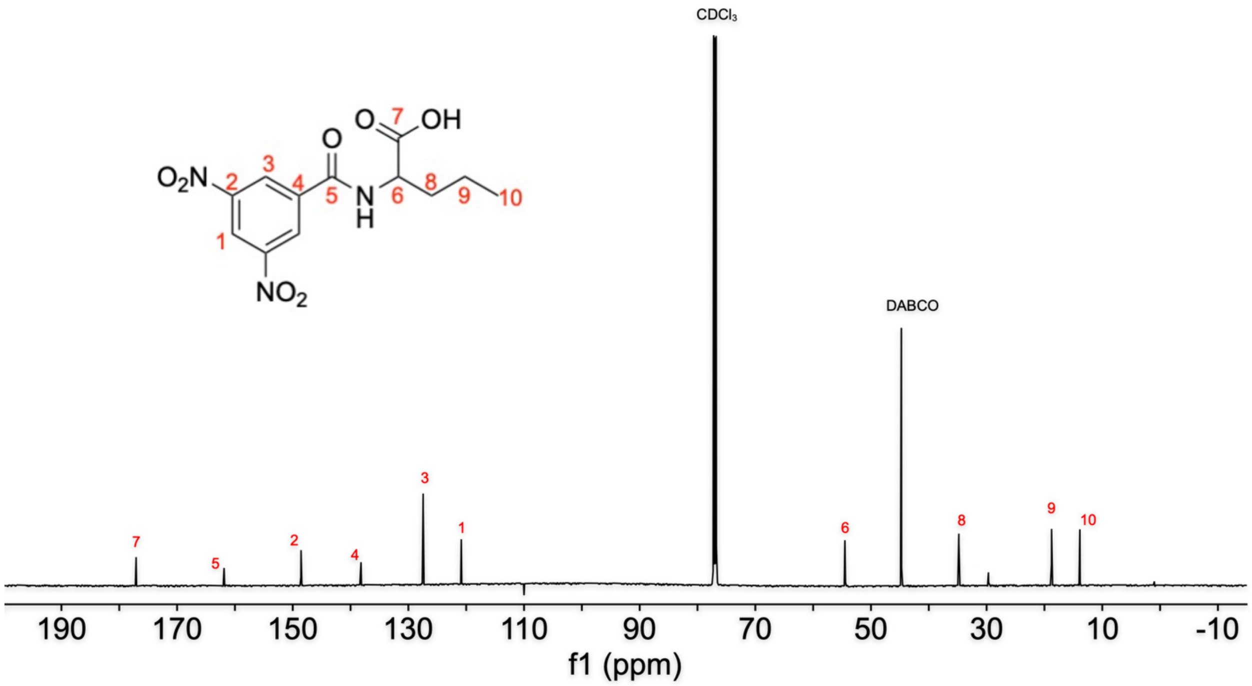

Figure S3. $^{13}C\{^{1}H\}$ NMR (150 MHz, $CDCl_3$, 25 °C) spectrum of **6** (15 mM) in the presence of 1 equiv of DABCO.

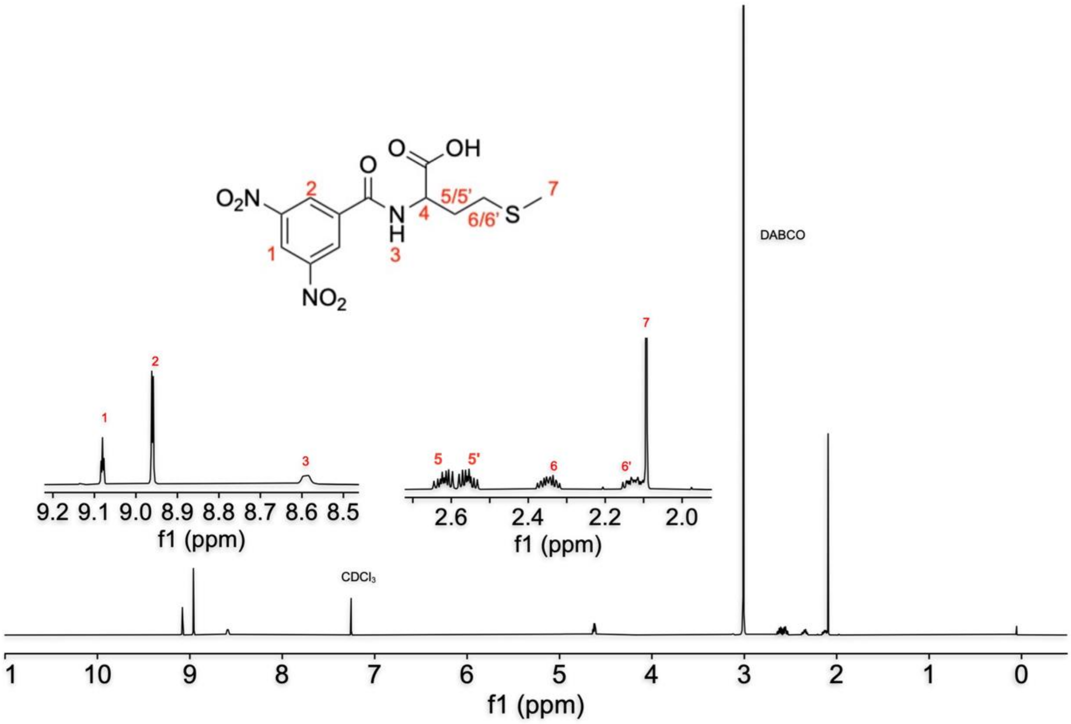

Figure S4. ^{1}H NMR (600 MHz, $CDCl_3$, 25 °C) spectrum of **7** (15 mM) in the presence of 1 equiv of DABCO.

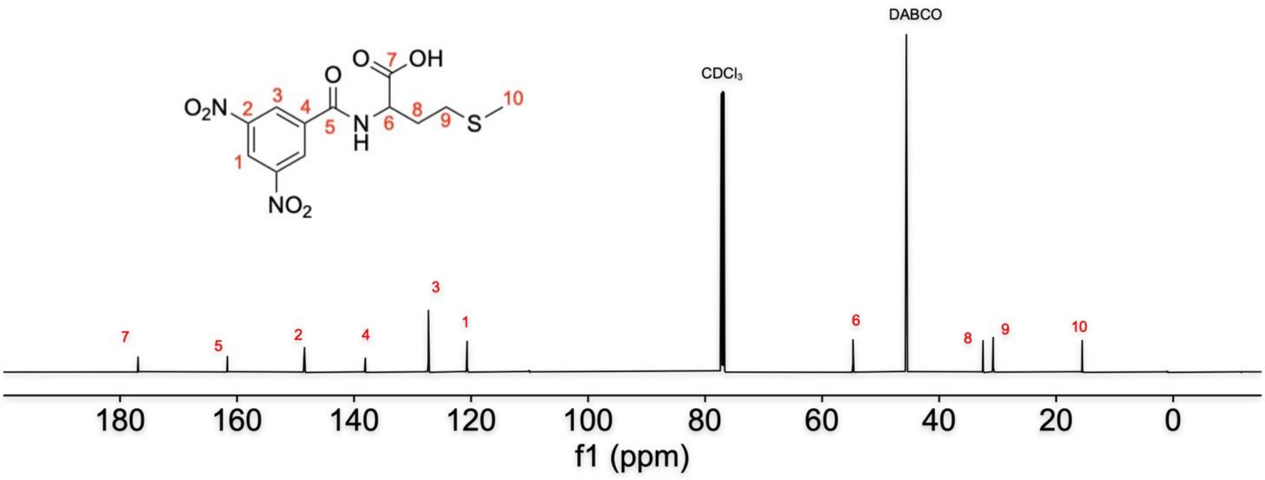

Figure S5. $^{13}C\{^{1}H\}$ NMR (150 MHz, $CDCl_3$, 25 °C) spectrum of **7** (15 mM) in the presence of 1 equiv of DABCO.

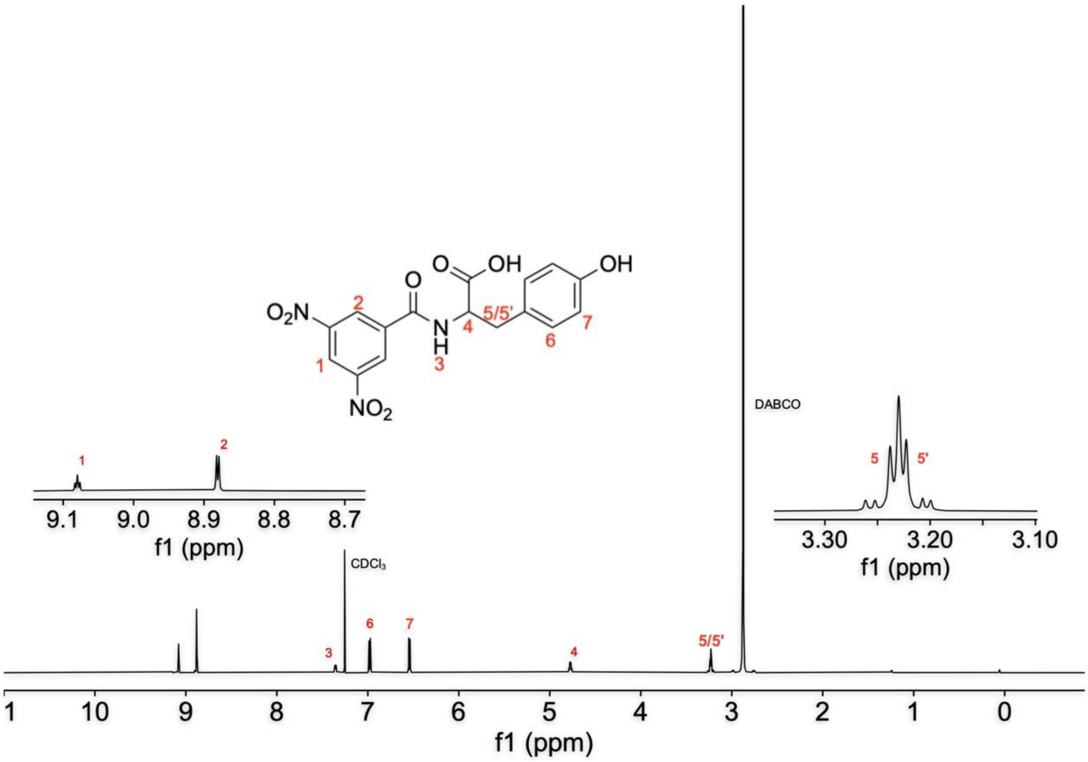

Figure S6. ^{1}H NMR (600 MHz, $CDCl_3$, 25 °C) spectrum of **8** (15 mM) in the presence of 1 equiv of DABCO.

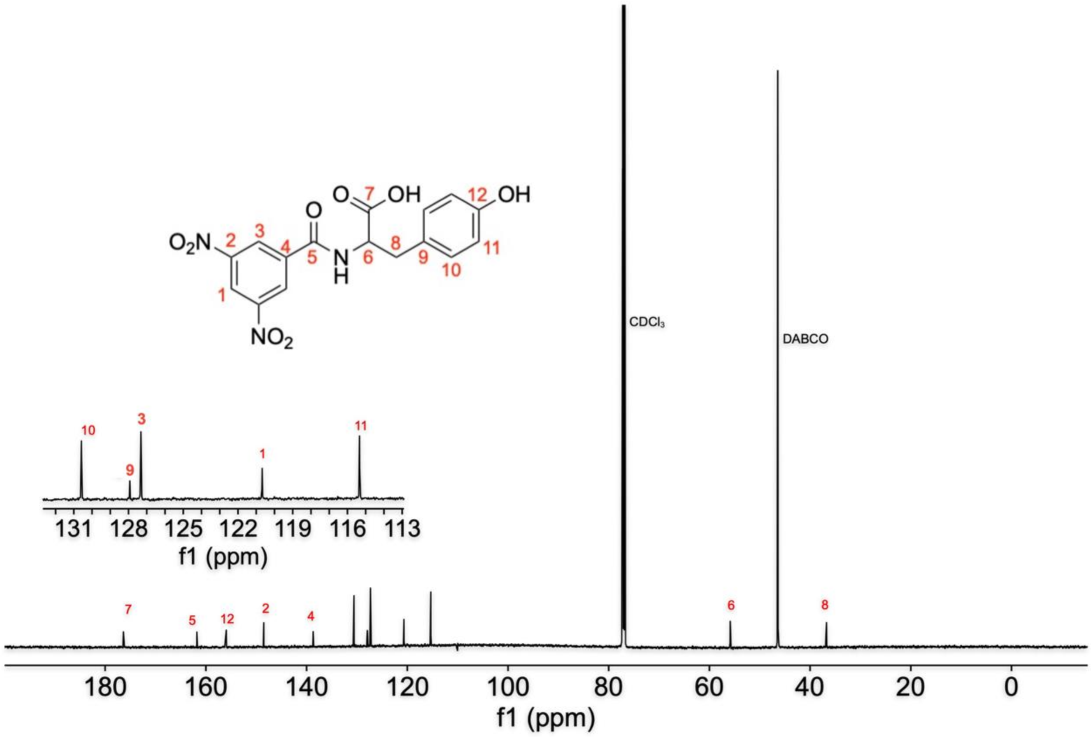

Figure S7. $^{13}C\{^1H\}$ NMR (150 MHz, $CDCl_3$, 25 °C) spectrum of **8** (15 mM) in the presence of 1 equiv of DABCO.

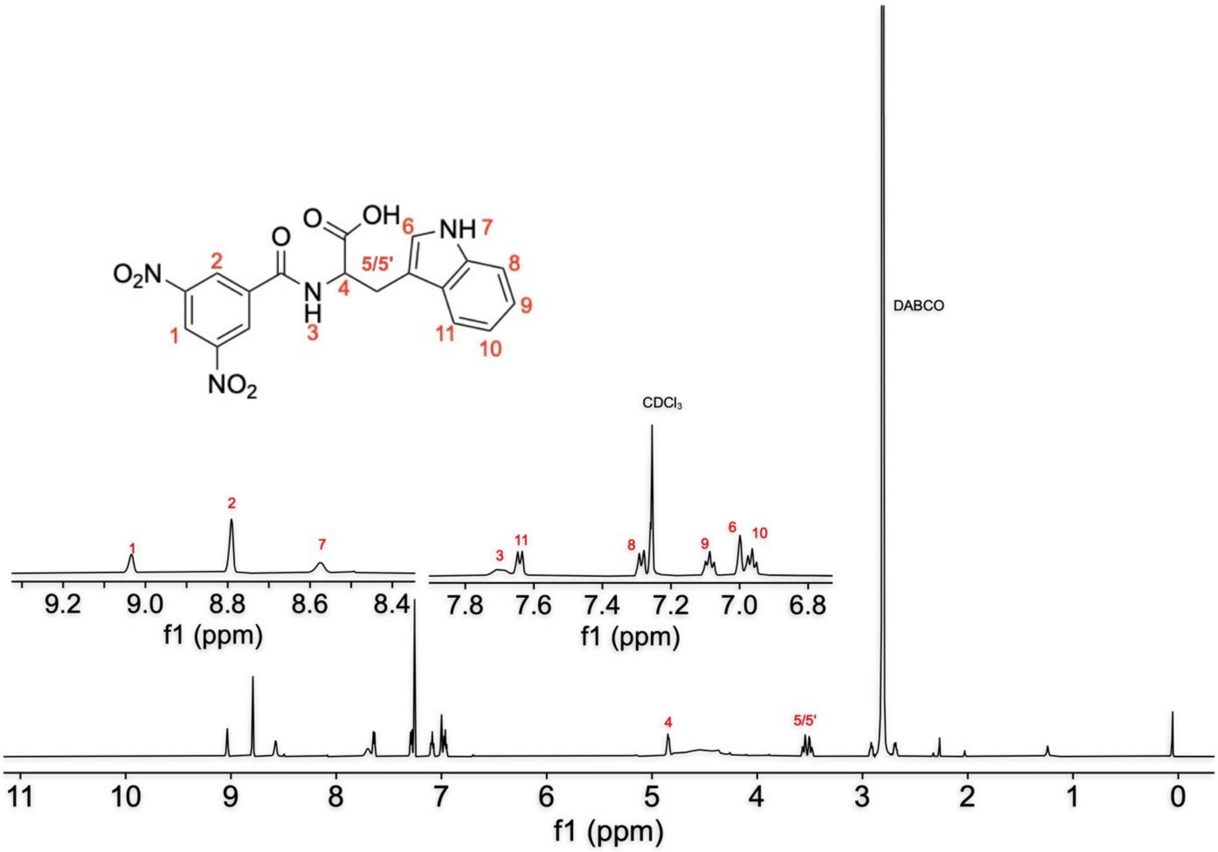

Figure S8. ^1H NMR (600 MHz, $CDCl_3$, 25 °C) spectrum of **9** (15 mM) in the presence of 1 equiv of DABCO

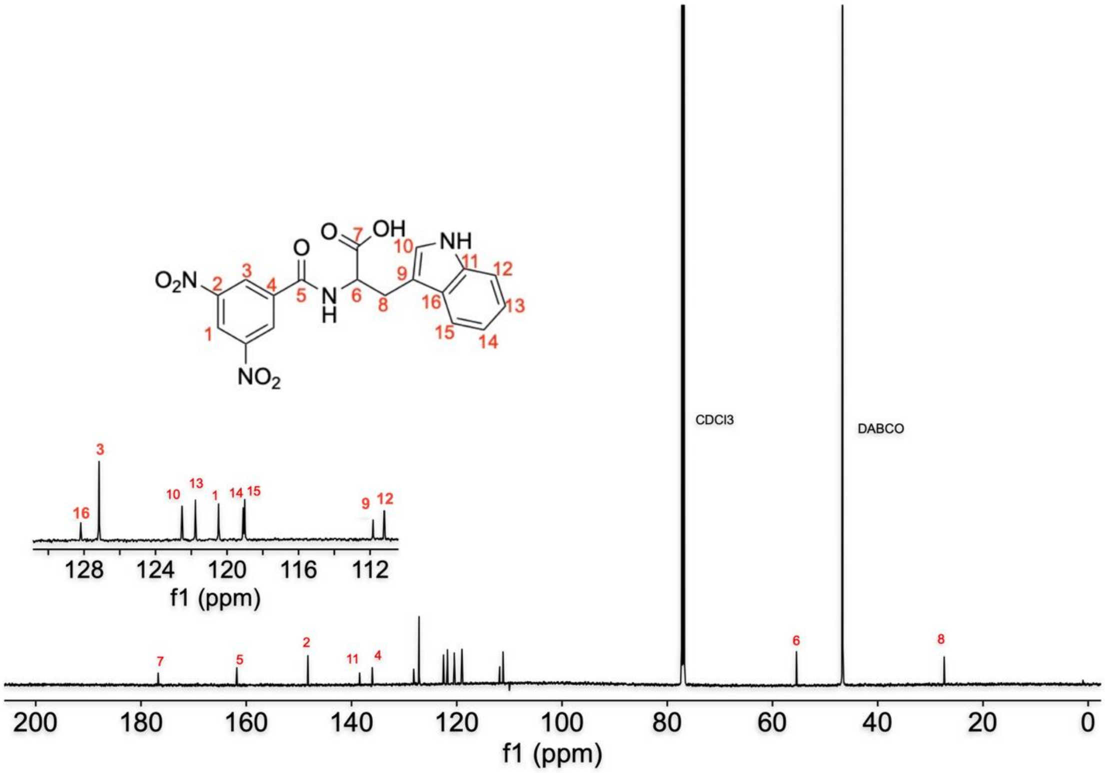

Figure S9. $^{13}C\{^1H\}$ NMR (150 MHz, 25 °C, $CDCl_3$) spectrum of **9** (15 mM) in the presence of 1 equiv of DABCO.
